# Socially disadvantaged city districts show a higher incidence of acute ST-elevation myocardial infarctions with elevated cardiovascular risk factors and worse prognosis

**DOI:** 10.1186/s12872-017-0683-2

**Published:** 2017-09-22

**Authors:** J. Schmucker, S. Seide, H. Wienbergen, E. Fiehn, J. Stehmeier, K. Günther, W. Ahrens, R. Hambrecht, H. Pohlabeln, A. Fach

**Affiliations:** 1 The Bremer Institut für Herz- und Kreislaufforschung (BIHKF) am Klinikum Links der Weser, Bremen, Germany; 2 The Leibniz-Institut für Präventionsforschung und Epidemiologie Bremen – BIPS, Bremen, Germany

## Abstract

**Background:**

The importance of socioeconomic status (SES) for coronary heart disease (CHD)-morbidity is subject of ongoing scientific investigations. This study was to explore the association between SES in different city-districts of Bremen/Germany and incidence, severity, treatment modalities and prognosis for patients with ST-elevation myocardial infarctions (STEMI).

**Methods:**

Since 2006 all STEMI-patients from the metropolitan area of Bremen are documented in the Bremen STEMI-registry. Utilizing postal codes of their home address they were assigned to four groups in accordance to the Bremen social deprivation-index (G1: high, G2: intermediate high, G3: intermediate low, G4: low socioeconomic status).

**Results:**

Three thousand four hundred sixty-two consecutive patients with STEMI admitted between 2006 and 2015 entered analysis. City areas with low SES showed higher adjusted STEMI-incidence-rates (IR-ratio 1.56, G4 vs. G1). This elevation could be observed in both sexes (women IRR 1.63, men IRR 1.54) and was most prominent in inhabitants <50 yrs. of age (women IRR 2.18, men IRR 2.17). Smoking (OR 1.7, 95%CI 1.3–2.4) and obesity (1.6, 95%CI 1.1–2.2) was more prevalent in pts. from low SES city-areas. While treatment-modalities did not differ, low SES was associated with more extensive STEMIs (creatine kinase > 3000 U/l, OR 1.95, 95% CI 1.4–2.8) and severe impairment of LV-function post-STEMI (OR 2.0, 95% CI 1.2–3.4). Long term follow-up revealed that lower SES was associated with higher major adverse cardiac or cerebrovascular event (MACCE)-rates after 5 years: G1 30.8%, G2 35.7%, G3 36.0%, G4 41.1%, p (for trend) = 0.02. This worse prognosis could especially be shown for young STEMI-patients (<50 yrs. of age) 5-yr. mortality-rates(G4 vs. G1) 18.4 vs. 3.1%, *p* = 0.03 and 5-year-MACCE-rates (G4 vs. G1) 32 vs. 6.3%, *p* = 0.02.

**Conclusions:**

This registry-data confirms the negative association of low socioeconomic status and STEMI-incidence, with higher rates of smoking and obesity, more extensive infarctions and worse prognosis for the socio-economically deprived.

## Background

The relationship between socioeconomic status (SES) and disease development in industrialized nations is well established [1–3]. The SES, usually measured by parameters like income, educational status and occupation, can be defined as individual level SES by assessing the social status of each study participant or area level SES, reflecting the subjects‘ living neighbourhood. Although distinctness of the results may differ, both, individual SES and area level SES, have shown to be valid predictors of morbidity and mortality [4, 5]. Other studies differentiate between contextual and compositional effects on health, the latter emphasizing the influence of individual characteristics‘on disease, while the contextual approach focuses on the role of the patients‘ living environment. In previous studies an association of socioeconomic status and incidence as well as prognosis of cardiovascular disease (CVD) and myocardial infarctions has been observed [6–11]. For example Diez Roux et al. found an increased incidence of coronary heart disease associated with living in a disadvantaged neighbourhood with a hazard ratio (HR) of 3.1 among whites and 2.5 among blacks as compared to persons from most advantaged neighbourhoods [6]. In the Netherlands neighbourhood socioeconomic inequalities were observed in AMI incidence when comparing the most deprived with the most affluent neighbourhoods (RR for AMI was 1.34 in men (95% confidence interval (CI) 1.32 to 1.36) and 1.44 in women (95% CI 1.42 to 1.47) [10].

Recently a Canadian study group could show higher incidence of cardiovascular disease and rate of cardiovascular events (cardiovascular death, myocardial infarction, stroke and heart failure) in low-income countries compared to high- and middle-income countries [12]. In addition income ratios seem to have an influence on life expectancy. An actual investigation came to the result, that higher income was associated with greater longevity throughout the income distribution. Between the richest 1% and poorest 1% of individuals, life expectancy varied between 14.6 years (95% CI, 14.4 to 14.8 years) for men and 10.1 years (95% CI, 9.9 to 10.3 years) for women [13].

Several studies also described a negative impact of SES on treatment of patients with ST-elevation myocardial infarction (STEMI) [14–16], while others did not [17].

Since all incident cases of STEMI of residents of the city of Bremen are admitted to the Bremen heart center we are able to perform a comprehensive epidemiological analysis on a well defined clinical entity. We analysed the impact of socioeconomic status on incidence for STEMI-patients admitted between 2006 and 2015. Patients were assigned to SES-groups through the postal codes of their place of residence. Furthermore the influence of area level SES on the cardiovascular risk profile, infarction-severity and short- and long-term outcome after STEMI was analysed.

## Methods

### The Bremen STEMI-registry (BSR)

The BSR is a monocentric prospective registry of patients admitted with STEMI at the Bremen heart center. The Bremen heart center is exclusively responsible for 24-h-PCI-service in a large region in northwest Germany, serving a population of more than 800.000 residents from Bremen and in the northwestern part of Lower Saxony. Emergency services and regional hospitals are connected by telephone and fax for announcement of urgent coronary catheterization in case of STEMI. Since all STEMIs from the Bremen heart center are documented in the BSR it claims to give a complete statistical overview about the clinical course (short- and long-term) of STEMI-patients in the region of Bremen. The BSR was established in 2006 and is still running. Documentation is accomplished via data sheets completed by the responsible interventional cardiologist and/or through patient records after a physician has confirmed the exact diagnosis. Data about age, residence, sex, temporal delays of treatment, concomitant diseases, severity of STEMI and acute medical or interventional treatment are recorded. At discharge major adverse cardiac and cerebral events are documented. Follow-up examination is performed after 1 and 5 years by a telephone interview. Further details have been published previously [18].

### Definition of STEMI and disease severity

STEMI was defined as persistent angina pectoris for ≥20 min in conjunction with a ST-segment elevation in two contigous leads of ≥0.25 mV in men below the age of 40 years, ≥0.2 mV in men over the age of 40 years, or ≥0.15 mV in women in leads V2-V3 and/or ≥0.1 mV in all other leads or new left bundle branch block [19].

Disease severity was evaluated using surrogate parameters, such as acute heart failure, pulmonary edema or cardiogenic shock at admission by the Killip-classification, multivessel disease with or without left-main-lesion and by measurement of the post-AMI ejection fraction. The peak creatine kinase (CK) was used to assess extension of myocardial infarction with laboratory controls routinely performed in STEMI-patients once to twice daily. To assess the quality of care, door-to-balloon time, interventional procedures and medication at hospital discharge were analysed.

### The Bremen social deprivation index

The Bremen social deprivation index (SDI) was developed in 1993 to describe social inequalities in the city of Bremen in Germany. In its most recent update (2009) a ranking was established for 89 city districts, using census and public data on education, voting participation, unemployment, crime statistics and immigration flow. A positive composite index indicates a socially privileged, a negative composite index a socially disadvantaged city district with an overall mean SDI of 0 [20]. Since only postal codes were available in the BSR the SDIs of the 89 city districts were aggregated to 33 postal code regions of Bremen. Population data stem from municipial data of 2008.

### Statistical analysis

Since a normal distribution of the social deprivation index across the postal code regions could be assumed (Kolgmorov-Smirnov-test of the 33 postal code regions: *p* = 0.15) distribution into groups was made according to the mean and standard deviation of the SDI: Within one standard deviation of the mean SDI postal codes were assigned to the groups G2 (social status: intermediate high) and G3 (social status: intermediate low). Postal codes with an SDI below −1 SD of the mean were assigned to G4 (social status: low), postal codes above +1 SD of the mean SDI were assigned to G1 (social status: high). Baseline characteristics of STEMI-patients were given as mean values ± standard deviation for continuous variables (age, left ventricular ejection fraction, door to balloon-time, number of coronary vessels affected) and in absolute numbers and percentages for categorical variables: (diabetes, obesity (body mass index > 30 kg/m^2^), smoking, multivessel-disease, prehospital cardiopulmonal resuscitation (CPR), peak CK > 3000 U/l, acute heart failure, positive family history (FH) for CAD/CVD, primary PCI, CABG, LVEF ≤ 40%, medication at admission and at discharge) by using SAS/STAT (version 9.3., 2011). Statistical significance was tested using Chi-square for categorical and t-tests for continuous variables. For comparison of the groups ANOVA tests were used for continuous variables, Chi-square for trend-test (Cochrane-Armitage) for categorical variables. A *p*-value <0.05 was considered statistically significant. Sex-, age-, and SES-group-specific incidence rates (per 100.000 persons/year) were estimated as the sum of of STEMI-events during 2006–2015 divided by the sum of persons at risk in the strata of sex, age- and SES-group. Incidence rate ratios (IRRs) and 95% confidence intervals were estimated by log-linear Poisson regression of sex-, age- and SES-group-specific number of STEMI-events with logarithm of persons at risk as offset. Multivariate logistic regression analysis were performed with SES-groups, age, sex and prior CVD as the independent variable and age, sex, smoking, obesity, diabetes mellitus, CK > 3000 U/l, primary PCI, successful PCI and LVEF < 40% as the dependent variable. Analysis of mortality and adverse-events-rates (major adverse cardiac and cerebrovascular events (MACCE): death, stroke, myocardial reinfarction) was performed after stratifying for age (<50 yrs., ≥50 yrs.).

## Results

### Study population

The total study population comprised the city of Bremen with approximately 550,000 inhabitants. Between January 1st 2006 and December 31th 2015 a total of 3462 residents of the city of Bremen a STEMI was newly diagnosed. They were assigned to groups (G1 to G4) according to the social deprivation index of their home address, with G1 representing the most affluent neighbourhoods and G4 representing the most deprived ones (Table 1).

**Table 1:** Sociodemographic baseline characteristics of SES-groups

	G1 (high SES)	G2 (intermediate high SES)	G3 (intermediate low SES)	G4 (low SES)
% of total population	19	24	43	14
Inhabitants <19 yrs. of age (%)	14	13	15	19
STEMIs 2006–2016 (n)	519	813	1554	576
SDI by definition	≥56	0 to 56	−56 to 0	≤ −56
SDI range	99 to 56	50 to 1	−3 to −50	−59 to −121
Mean SDI±SD	78 ± 16	26 ± 18	−31 ± 16	−86 ± 29

### Incidence of STEMI

**Fig. 1:**
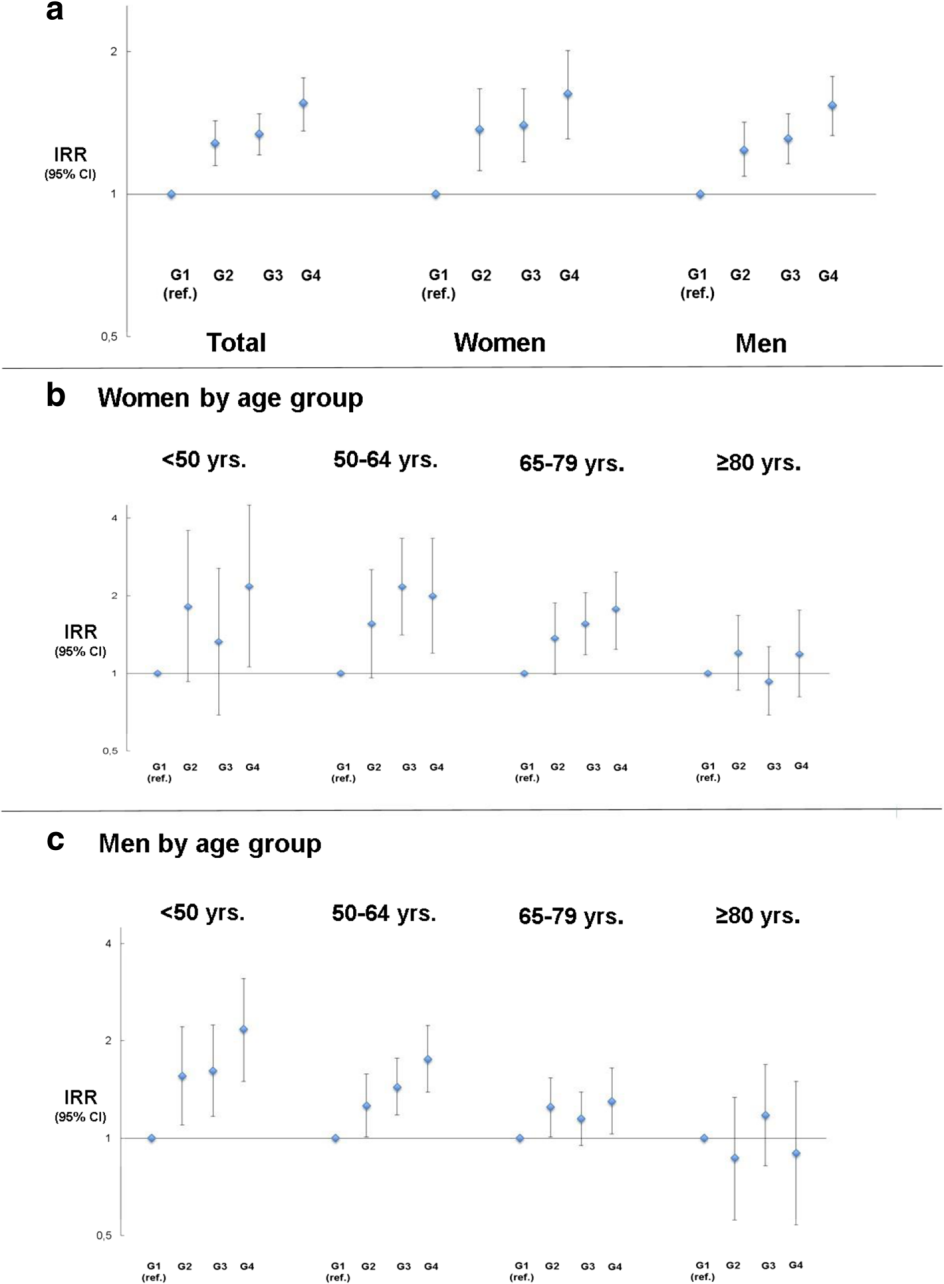
Incidence rate ratios by SES. Adjusted incidence rate ratios (IRRs) with 95% CI, calculated for SES-class G2 to G4 with G1 as baseline (1). Calculation based on total numbers (**a**) or prestratified by age for women (**b**) and men (**c**)

The unadjusted incidence-rate (per 100.000 persons/year) of STEMI in the city of Bremen between 2006 and 2015 was 63 per 100,000 inhabitants/year or 74 per 100,000 inhabitants/year when excluding inhabitants <18 yrs. from the calculation model (Table 2). The hypothesis, that the number of STEMIs was equally distributed over time could not be rejected for any of the four SDI-Groups (*p*-values calculated by means of a chi-square goodness-of-fit test): G1 (*p* = 0.45), G2 (*p* = 0.14), G3 (p = 0.14), G4 (*p* = 0.10). A rising incidence of STEMI could be observed with declining SDI category. Adjusted relative risk (for age and sex) for STEMI showed a 56% increase of STEMI-incidence among pts. from low SES neighbourhoods as compared to the more affluent city regions. The association of rising STEMI incidence with increased social deprivation could be demonstrated in both sexes (Fig. 1a). The socioeconomic gradient was most prominent in young patients (<50 years of age) showing a 2.18 fold elevation of STEMI-incidence when comparing G4 to G1 in women (Fig. 1b) and a 2.17 fold elevation in men (Fig. 1c). In the older ages groups this gradient consistently decreased until being no longer significant in pts. ≥ 80 years for men and women alike (Fig. 1b and c).

**Table 2:** Population ≥ 18 yrs. of age at risk and STEMI-events 2006–2015

		G1	G2	G3	G4
All STEMIs	Population at risk	87,783	114,107	200,548	64,087
	Events ^a^	519	813	1554	576
	Incidence rate (IR/ 95%CI)	59 (54–65)	71 (66–76)	77 (74–81)	90 (83–98)
Men	Population at risk	41,345	58,548	102,460	32,357
	Events ^a^	352	576	1097	407
	Incidence rate (IR/ 95%CI)	85 (77–95)	98 (91–107)	107 (101–114)	126 (114–139)
Women	Population at risk	46,438	55,559	98,088	31,730
	Events ^a^	167	237	457	169
	Incidence rate (IR/ 95%CI)	36 (31–42)	43 (37–48)	47(42–51)	53 (46–62)
Age 18–49	Population at risk	42,972	61,834	104,873	33,326
	Events ^a^	57	139	229	100
	Incidence rate (IR/ 95%CI)	13 (10–17)	22 (19–27)	22(19–25)	30 (24–36)
Age 50–64	Population at risk	19,873	26,569	44,815	14,861
	Events ^a^	143	267	533	203
	Incidence rate (IR/ 95%CI)	72 (61–85)	100 (89–113)	119(109–130)	137 (118–157)
Age 65–79	Population at risk	18,171	18,524	37,191	11,863
	Events ^a^	216	293	579	208
	Incidence rate (IR/ 95%CI)	118 (103–136)	158 (141–177)	156(143–168)	175 (154–202)
Age > 79	Population at risk	6767	7180	13,669	4037
	Events ^a^	103	114	213	65
	Incidence rate (IR/ 95%CI)	152 (124–185)	159 (131–191)	156(137–178)	161 (124–205)

^a^STEMIs 2006–2015

### Baseline characteristics of STEMI-patients

**Fig. 2:**
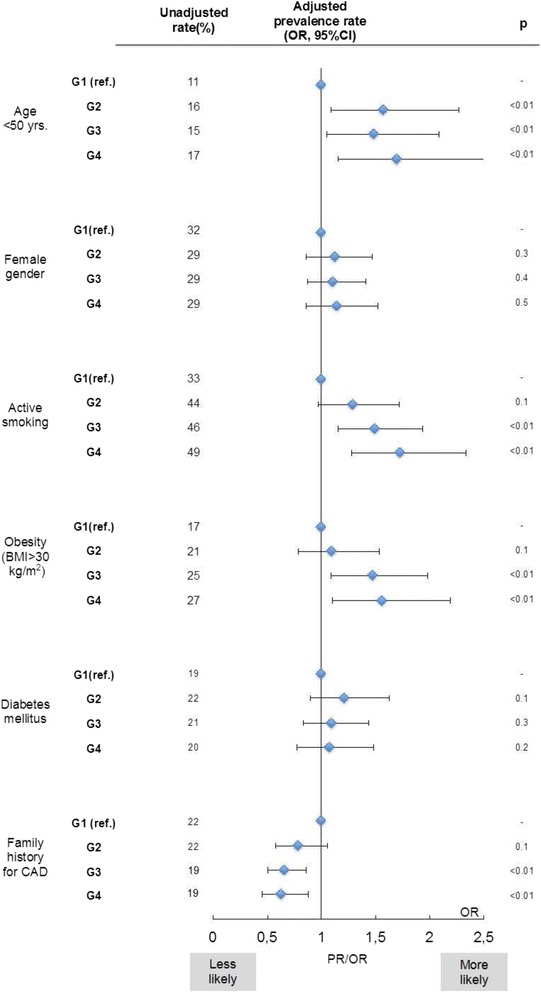
Association of SES with age, gender and cardiovascular risk profile. Impact of SES-class (G2-G4, G1 = baseline) on prevalence of young age, female gender and cardiovascular risk factors in STEMI-patients. OR calculated with multivariate adjustment for age, gender and known cardiovascular disease

The 3462 patients admitted with STEMI between 2006 and 2015 were on average 64.8+/−13 years old, 71% were male, 44% were active smokers, 21% had diabetes and 23% were obese (BMI > 30 kg/m^2^). Patients from neighbourhoods with a lower SDI were on average younger at time of STEMI, more likely to smoke and more likely to be obese than those from more affluent city districts. In contrast the prevalence of a positive family history for CAD was significantly lower in patients from low-SES city districts. Gender distribution as well as prevalence of diabetes in STEMI patients did not differ substantially between SDI categories (Table 3). A multivariate analysis confirmed these results: higher degrees of social disadvantage was positively associated with higher likelihood of suffering the STEMI at young age, to smoke or to be obese at time of STEMI. In contrast social deprivation and the prevalence of positive family history for CAD were inversely related while gender and diabetes mellitus did not show significant differences in prevalence between SDI-categories (Fig. 2).

**Table 3:** Cardiovascular risk profile of STEMI-patients according to SES-group

	G1 (*n* = 519)	G2 (*n* = 813)	G3 (*n* = 1554)	G4 (*n* = 576)	p-value
Age (yrs) ± SD	67.6 ± 13	64.1 ± 13	63.1 ± 13	63.1 ± 13	<0.01
Female Gender (%)	31.8	29.1	29.4	29.3	0.08
Smoking (%)	33.3	44.2	45.9	49.1	<0.01
Mean BMI (kg/sqm) ± SD	26.7 ± 5	27.3 ± 5	27.6 ± 5	27.9 ± 5	<0.01
BMI > 30 kg/sqm (%)	17.0	21.4	25.2	26.8	<0.01
Diabetes mellitus (%)	18.8	21.9	20.5	20.4	0.3
Family history (FH) for CAD (%)	22.2	22.3	18.6	18.5	0.02
Known CV disease (%)	23.8	24.8	27.5	24.6	0.9

### Severity of STEMI

**Fig. 3:**
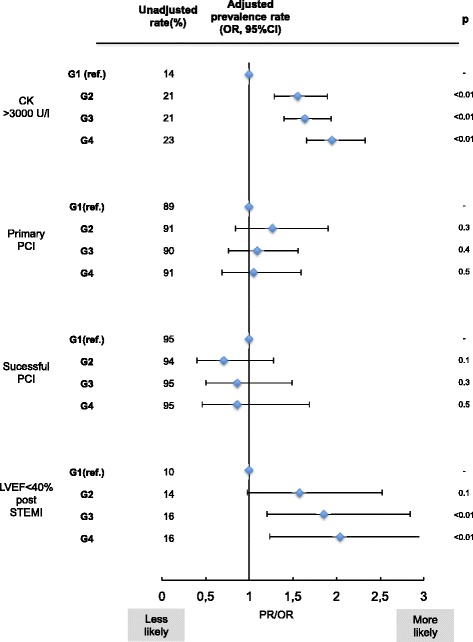
Association of SES-group on infarction severity and treatment modalities. Impact of SES class (G2-G4, G1 = baseline) on prevalence of CK > 3000 U/l, primary/successful PCI and LVEF < 40% post MI for STEMI-patients. OR calculated with multivariate adjustment for age, gender and known cardiovascular disease

While the prevalence of multi-vessel-disease did not differ substantially between SES-classes, pts. from low-SES city districts showed greater extension of STEMI, measured by the peak creatine kinase (peak CK) and had on average lower left-ventricular-ejection-fraction (LVEF) post-STEMI (Table 4). The relation between social deprivation and infarct size and subsequent LV-EF-reduction post STEMI remained significant in a multivariate model (Fig. 3). Rates of prehospital resuscitation as well as the rate of cardiogenic shock in STEMI patients did not differ between SES-classes (Table 4).

**Table 4:** Severity of CAD/ STEMI, treatment modalities and outcome according to SES-group

	G1 (n = 519)	G2 (n = 813)	G3 (n = 1554)	G4 (n = 576)	p-value
Severity of myocardial infarction					
Number of coronary vesssels affected ±SD	1.99 ± 0.8	1.94 ± 0.8	1.91 ± 0.8	1.84 ± 0.8	0.09
Cardiogenic shock (%)	12.1	13.1	12.0	12.4	0.8
Prehospital CPR (%)	10.9	11.3	10.8	10.3	0.5
Subacute MI(%) ^a^	19.3	23.9	20.5	19.9	0.8
Mean peak CK (U/l) ± SD	1599 ± 1679	1838 ± 2019	1913 ± 2188	2025 ± 2167	<0.01
Peak CK > 3000 U/l (%)	13.7	20.5	20.9	23.3	<0.01
Post AMI LVEF in % ± SD	49.6 ± 9	48.9 ± 10	48.4 ± 11	47.5 ± 10	<0.01
LVEF <40% (%)	10.4	13.8	15.8	16.3	0.01
Treatment modalitites					
Primary PCI (%)	89.0	91.1	89.9	90.9	0.9
CABG (%)	6.1	3.9	4.8	3.4	0.14
Conservative therapy regimen (%)	4.8	4.9	5.2	5.7	0.8
Door to balloon time in min (median ± SD)	48 ± 32	43 ± 44.5	46 ± 40	44 ± 41	0.74
GP2b3a-Inhibitors (%)	70	67	66	70	0.7
ASA (at discharge) (%)	94	96	94	95	0.64
ADP-Antagon. (ad) (%)	90	90.5	90	94	0.23
Beta-blockers (ad) (%)	83	84	80	84	0.25
Statins (ad) (%)	86	87	87	86	0.9
ACE/ATR-Inhibitors (ad) (%)	77	80	79	79	0.9
Outcome					
Mortality <72 h (%)	3.9	3.6	3.9	4.2	0.71
Inhospital mortality (%)	8.8	7.6	7.9	8.1	0.71
1-year-mortality (%)	15.7	15.1	14.9	16.3	0.9
1-year-MACCE (%)	19.2	19.5	19.1	20.4	0.8
5-year-mortality (%)	23.9	27.7	25.7	28.3	0.46
5-year-MACCE (%)	30.8	35.7	36.0	41.1	0.02

^a^subacute MI was defined as onset of symptoms >12 h before admission to PCI- center and/or signs of subacute STEMI in initial ECG

### Treatment modalities

The proportion of patients receiving a successful primary PCI was >89%, with no significant disparities when comparing the socioeconomic groups (Fig. 3). Moreover, comparable proportions of STEMI-patients had early surgical revascularisation procedures (CABG) or a conservative therapy regime. Prior to the coronary angiography pts. from all SES-groups showed similar average door-to-balloon-times. Patients from all quartiles received additional Gp2b3A-antagonist-medication (Tirofiban or Abciximab) at a similar frequency. At discharge from hospital there was no difference in standard medication (ASA, ADP-antagonists, beta blockers, statins, ACE/ATR-Inhibitors) between the groups (Table 4).

### Impact of SES on prognosis

**Fig. 4:**
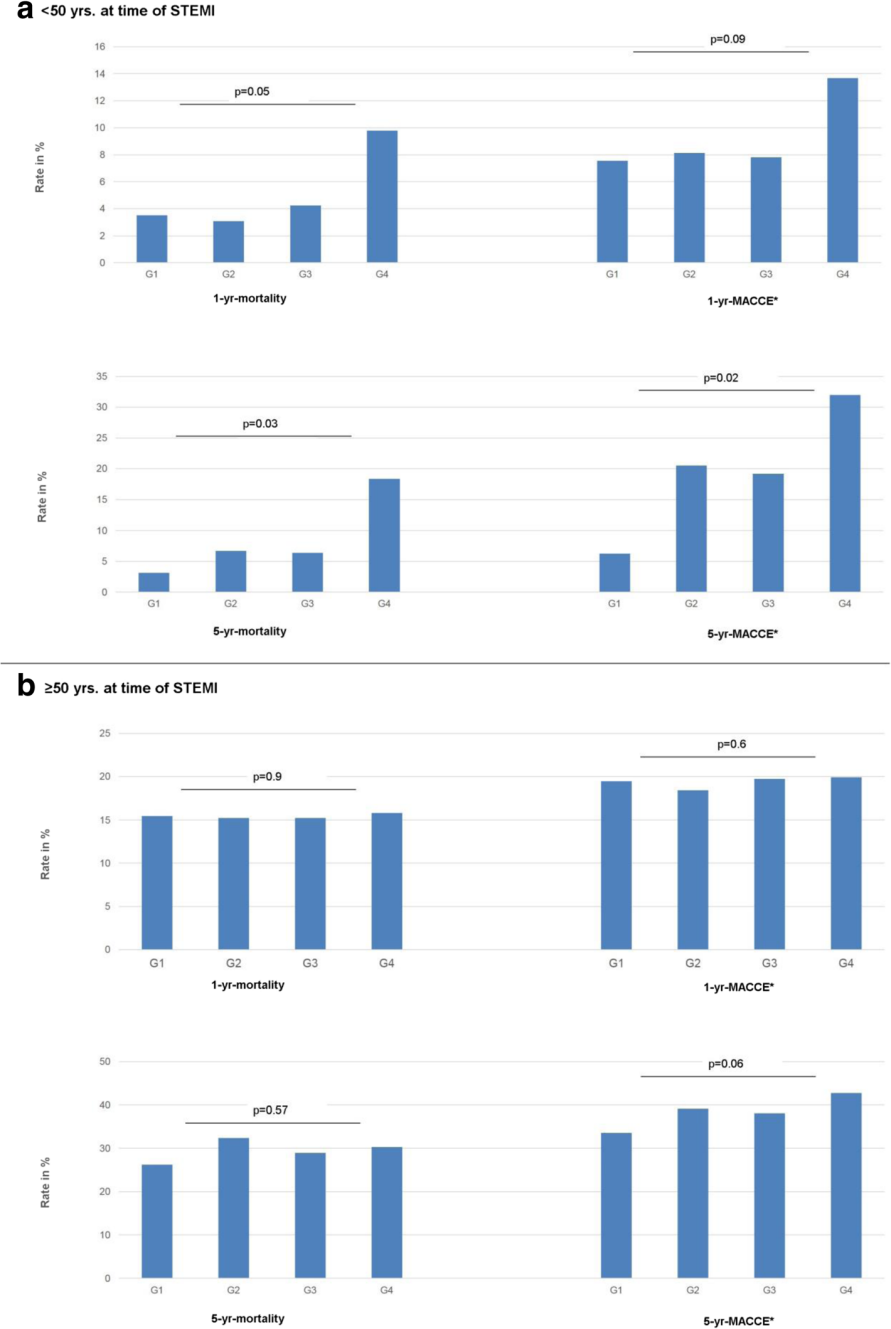
Impact of SES-group on long term outcome after STEMI. Impact of SES-group on long term-outcome after STEMI, patients stratified by age (<50 yrs. (**a**), ≥50 yrs. (**b**)). P calculated with Chi-square for trend. *MACCE: death, stroke, reinfarction

While SES had no significant impact on inhospital-mortality, 1-year-mortality, 1-year-MACCE-rates and 5-year-mortality-rates, lower SES was associated with a significant increase in MACCE-events within 5 years after STEMI, showing a 34% increase in event rates when comparing pts. from the most socially deprived city areas to those from the city areas with the highest SES (Table 4). When stratifying follow-up-data by age (<50 yrs. of age and ≥50 yrs. of age) a negative impact of social deprivation on long-term-outcome could be shown for the young STEMI-cohort with elevated 1-year mortality-, 1-year-MACCE-rates as well as 5-year-mortality- and 5-year-MACCE-rates for the socially deprived (Fig. 4a). In contrast short-term- (<72 h) (G1: 1.3%, G2: 0.9%, G3 1.0%, G4:1.6%; p (for trend) = 0.8) and in-hospital mortality-rates (G1: 2.6% G2: 2.4%, G3: 3.9%, G4: 3.6%, p (for trend) = 0.1) were not significantly affected by SES-class. For pts. ≥ 50 yrs. SES did not show an impact on 1- and 5-year mortality-and MACCE-rates, while overall event-rates were generally higher due to the advanced patients' age (Fig. 4b).

## Discussion

The present data of the Bremen STEMI registry show a significant association between socioeconomic status and incidence of STEMI. This negative association could be observed most strikingly for the young with a 2.2 fold higher risk for inhabitants younger than 50 years for suffering a STEMI when living in low SES city areas. Patients with STEMI from socially disadvantaged city districts were more likely to smoke and to be obese. Neither in-hospital treatment nor short-term mortality showed significant differences between the different SES-groups. However patients living in low-SES city-districts were more likely to suffer severe myocardial infarctions measured by peak-CK and showed higher rates of severe left-ventricular-impairment (EF < 40%) post-STEMI. Evaluation of long-term-prognosis showed higher 5-year-MACCE-rates for STEMI-patients from socially deprived city districts. The association of social deprivation and elevated mortality and MACCE-rates was most prominent for young-STEMI-populations showing a 3 fold elevation in 1-year-mortality, a 5.9 fold elevation in 5-year mortality and a 4.9 fold elevation in 5-year-MACCE-rates when comparing patients younger 50 yrs. from the city districts with the lowest SES to those from the most affluent ones.

Our findings are based on area-level-SES. Effects of socioeconomic inequity might therefore be attenuated and would be even more pronounced if individual-level socioeconomic data had been available. Whether the higher incidence and severity of STEMI as well as the poorer outcome of STEMI-pts. in the G4 group are attributable to contextual and/or compositional effects of the place of residence cannot be differentiated by our results but might be subject to latter investigation.

### Association of socioeconomic status and prevalence of CAD /incidence of AMI

An association between socioeconomic deprivation and increased risk for suffering from CAD and AMI has been described by prior investigations [10, 21]. Besides the observation of an increased risk for coronary events in socioeconomic disadvantaged neighbourhoods in four US study sites by Diez Roux [21], a social down-gradient of STEMI incidence could be observed in both sex groups and most significantly in young inhabitants in a Dutch study (relative risk in women 1.44, in men 1.34, decreasing social gradient with increasing age) [10]. These results are in good accordance to our data.

### Influence of SES on the cardiovascular risk profile for STEMI-patients

The elevated cardiovascular risk profile in patients with a low SES, in particular the higher rates of smokers and obese pts., might partly explain the elevated STEMI incidence. The association of active smoking and obesity with CAD and CAD-related mortality has been proven [22–25] and the influence of tobacco smoking in public areas on non-smokers` risk for AMI has been demonstrated by recent investigations [18]. Additionally there is some evidence, that beyond the classic risk factors for CAD the SES may have biological, behavioral and psychosocial components affecting the deprived [7, 26, 27]. Chetty et al. found that life expectancy more likely depends on health behaviors (i.e. smoking, obesity, exercise) rather than access to medical supplies or environmental factors (i.e. air pollution) [13]. In concern of CVD and MI disease determinants are varied. One aspect could be the neighbourhood in which people live, but social influences on health are complex and seem to operate through many different processes. Worldwide debate about to what extend contextual and/or compositional effects account for higher morbidity and mortality of the underprivileged is ongoing [28].

### Impact of SES on quality of care and treatment modalities

With respect to quality of care (door-to-balloon-times, PCI rates and medication) as well as with respect to short-term outcome there were no significant differences between the four SES groups. These results are in contrast to findings from Finland and the US, where low-income pts. received fewer therapy with antiplatelets, beta-blockers, lipid-lowering medication [13, 29] and reperfusion therapy [13]. In addition obtaining similar invasive procedures in treatment of STEMI was unlikely for ethnic minority members in the US, where racial disparities led to lower rates of primary PCI and longer door-to-balloon times in Afro-Americans [30, 31]. Alter et al. reported a negative association of SES and access to cardiac services, leading to elevated long -term mortality for patients from low SES areas even for Canada with its universal health care system [32]. Differences in findings about treatment strategies within different countries might still be attributed to altering health care systems. The fact, that there is no striking inequity in acute therapy and medication between the social groups in Bremen suggests, that the German health care system provides a guideline conform treatment for STEMI pts. independent of their social background.

### Impact of SES on severity of myocardial infarction, post-AMI-cardiac performance and short-term-mortality

Data from the BSR showed that living in low SES areas was associated with more severe myocardial infarctions, measured by the peak creatine kinase (CK) and higher rates of severe impairment of left ventricular ejection fraction post-STEMI. While Gerber et al. [33] showed higher rates of infarction-associated left-heart-failure for patients with low SES the impact of socioeconomic status on extent of MI has, to our knowledge, not been described before. However our data does not offer an explanation for the greater extent of MIs for the socially deprived since pre- as well as in-hospital-treatment modalities were similar across all SES-groups. While the rate of subacute MIs was similar in all SES-groups it however cannot be excluded, that the pain-to-alarm-interval, which is not coherently registered in this database, might have been longer for patients from socially deprived neighbourhoods.

### Social deprivation and long-term outcome after STEMI

Although acute treatment strategies were comparable between the different SES groups in the BSR, 5-year-MACCE-rates were significantly higher for STEMI-patients from low SES city areas. Stratification for age revealed, that the worse prognosis after STEMI was evident for young-STEMI patients showing a steep elevation in 1-year-mortality and 5-year mortality and MACCE-rates. This disadvantage could no longer be shown for elder patients. Similar findings with increased long-term mortality after AMI in pts. with lower SES have been described by prior studies [9, 32–38]. Winkleby et al. [38] demonstrated that 1-year case fatality from CAD was 1.6 times higher for women and 1.7 times higher for men in high versus low deprivation neighbourhoods. In Israel low income was shown to be associated with an increase in mortality risk when accompanied by low education among STEMI pts. (HR 2.64, 95% CI: 1.92–3.63) [34]. This disadvantage for STEMI-patients from underprivileged city areas regarding long-term outcome may be the result of poor treatment compliance especially in young STEMI patients, as previously low SES was found to be a predictor for non-adherence to post-discharge medication and lifestyle recommendations [39, 40]. Patients from low SES city areas were more likely to smoke at the index event. Since previous investigations have shown that the rate of smoking cessation after AMI is lower in the less educated [40], higher rates of remaining smokers even after STEMI should be assumed among the socially deprived patients contributing to their worse long-term-prognosis.

In total the elevated STEMI incidence and the higher long-term mortality in low SES city areas of Bremen may indicate a lack of efficacy of primary and secondary prevention strategies in those city districts. Consequently prevention programs should focus on socially disadvantaged neighbourhoods to further improve primary and secondary prevention strategies.

### Study limitations

Limited data on long-term medical adherence and risk-factor-adjustment of pts. after STEMI was available in the BSR, so that further studies are required to complete knowledge about adherence to secondary prevention strategies.

Due to the catchment area of the Bremen heart center, we are limited to a relative small study population, but on the other hand for the same reason we can give a comprehensive view of the clinical care of STEMI in Bremen, which minimizes bias due to regional differences in treatment strategies. Although the Bremen heart center is the only regional provider for emergency PCI in case of STEMI, we cannot rule out that single cases of STEMI were treated elsewhere, were not presented to receive emergency-PCI or died before admission to the cath-lab. This might have generated a selection bias.

Our database did not provide individual level SES. The analysis therefore relied on area level SES. This and the pooling from city district-SES to postal-code district SES to ultimately 4 SES-groups probably weakened the effect of social inequalities. However both, area level SES and individual level SES are valid for investigating the impact of socioeconomic status, only that the effects of area level SES are supposed to be weaker. Despite this, our study demonstrates a clear effect of SES on incidence and long-term-outcome, which might have been even more prominent if data on individual level SES had been available.

## Conclusions

In the present study a clear social gradient with an increased incidence of STEMI in socially deprived city districts could be observed. Patients with STEMI from socially disadvantaged city districts tend to be younger at time of infarction and are more likely to smoke and to be obese. The elevation in cardiovascular risk factors might partly explain the increased STEMI incidence. Patients from socially below average city districts furthermore showed larger myocardial infarctions and higher rates of severe impairment of cardiac function post STEMI.

While the social background does not seem to influence acute treatment modalities in STEMI nor short-term outcome, patients from low SES-city areas showed an elevation in 5-year-MACCE-rates. Which, besides pts.´ compliance and differences in infarction severity, may be the result of differences in secondary prevention strategies. The disadvantage in mortality and MACCE-rates however could especially be shown for STEMI-patients younger 50 years, where a 5.9 fold higher risk of death and a 4.9 fold elevation in MACCE-events within 5 years after STEMI could be observed.

Efforts and programs in primary and secondary prevention of CAD should therefore concentrate especially on city areas with a low socioeconomic profile.

## Abbreviations

ADP: Adenosine diphosphate
AMI: Acute myocardial infarction
ASA: Acetylsalicylic acid
ATR: Angiotensin receptor
BSR: Bremen STEMI registry
CABG: Coronary artery bypass graft
CAD: Coronary artery disease
CHD: Coronary heart disease
CI: Confidence interval
CVD: Cardiovascular disease
LMCA: Left main coronary artery
LVEF: Left ventricular ejection fraction
MI: Myocardial infarction
PCI: Percutaneous coronary intervention
Pts: Patients
PY: Person years
SD: Standard deviation
SDI: Social deprivation index
SEM: Standard error of mean
SES: Socioeconomic status
STEMI: ST elevation myocardial infarction
US: United States
Yrs: Years
